# The comparative study among different fractions of muscadine grape ‘Noble’ pomace extracts regarding anti-oxidative activities, cell cycle arrest and apoptosis in breast cancer

**DOI:** 10.1080/16546628.2017.1412795

**Published:** 2017-12-10

**Authors:** Jianming Luo, Shiren Song, Zheng Wei, Yu Huang, Yali Zhang, Jiang Lu

**Affiliations:** ^a^ Center for Viticulture and Enology, School of Agriculture and Biology, Shanghai Jiao Tong University, Shanghai, China; ^b^ The Viticulture and Enology Program, College of Food Science and Nutritional Engineering, China Agricultural University, Beijing, China; ^c^ Department of Food Science and Engineering, Jinan University, Guangzhou, Guangdong, China; ^d^ Guangxi Crop Genetic Improvement and Biotechnology Key Lab, Guangxi Academy of Agricultural Science, Guangxi, China

**Keywords:** Muscadine phenolic fraction, anti-oxidative activity, cell cycle arrest, apoptosis, cyclin, caspase

## Abstract

As a by-product of wine making, pomace contains rich amounts of phenolic compounds that can be potentially utilized as raw materials to make beneficial products especially for the anti-cancer agents including the breast cancer. *Muscadinia rotundifolia* ‘Noble’ is a wine-making grape cultivar, and to better use ‘Noble’ pomace, the most effective phenolic fractions in cancer inhibition must be identified. In this study, anti-oxidative activities of three separated fractions of ‘Noble’ pomace (F1, F2 and F3) were compared in 2,2-diphenyl-1-picrylhydrazyl and 2,2ʹ-azino-bis-(3-ethylbenzothiazoline-6-sulphonic acid) radical scavenging (DPPH and ABTS) assays as well as the ferric-reducing antioxidant power (FRAP) assay. The ability of different fractions to induce cell cycle arrest and apoptosis in MDA-MB-231 breast cancer cells was also evaluated by flow cytometry and Western blot analysis. Fraction F3, which contained a mixture of anthocyanidins and ellagic acids, exhibited the strongest anti-oxidative activity, as determined at both low and high concentrations in the DPPH and FRAP assays. F3 also demonstrated the greatest ability to induce apoptosis via caspase activation and cell cycle arrest by downregulating cyclin A and upregulating p21. F3 was thus the most effective bioactive fraction among those prepared from muscadine grape ‘Noble’ pomace.

## Introduction

Breast cancer, the leading cancer diagnosed in women worldwide, is also the most frequent cause of cancer mortality in many countries [1]. MDA-MB-231, one of the breast cancer cell lines commonly used in cancer research, normally has poor prognosis [2]. It is reported that, the combination treatment of phenolic compounds from plant such as resveratrol together with melphalan could inhibit MDA-MB-231 cells more efficiently [3]. Hence, natural phenolics had a great potential to be the anti-cancer agents.

*Muscadinia rotundifolia* Michx., also known as the muscadine grape, is indigenous to the southeastern region of the United States [4]. Previous studies have demonstrated that this grape confers multiple health benefits to humans, including cancer inhibition [5,6], a function that is primarily attributed to phenolic compounds [7]. Recently, several varieties of muscadine grapes were introduced to South China, and they adapted well to the climate [8]. One of these varieties, ‘Noble’, is a cultivar used for wine making [9,10]. As the by-product of wine-making, pomace still contains rich amounts of phenolic compounds. Thus, its use as the raw material is potentially beneficial for the production of health products especially for anti-cancer agents. The composition of pomace crude extract is complex, resulting in limited beneficial effects when directly applied. However, if the crude extract is fractionated and the most useful and effective phenolic fraction is identified, it would be possible to develop or improve downstream products utilizing only the most useful fraction. Thus, it is very important to identify the most effective phenolic fraction in a comparative study.

In previous reports, researchers found variable abilities to inhibit colon cancer among fractions from muscadine grape [11,12], although the analysis was limited only to the phenolic fractions or EA-rich fractions derived from fresh grape skin and/or pulp excluding extracts from seeds. On the other aspects, the anti-oxidative activity, the cell cycle arrest, and the induction of apoptosis in cancer cells were used to evaluate and compare the anti-cancer function exerted by different phenolic fractions [11].

To make better use of muscadine grape pomace as a raw material in production of health beneficial products or anti-cancer agents, it is necessary to evaluate and identify the most effective fraction by comparing their anti-oxidative activities, and abilities to induce cell cycle arrest and apoptosis. This study aims to identify this most effective phenolic fraction in MDA-MB-231 breast cancer cell inhibition isolated from muscadine grape ‘Noble’ pomace extracts.

## Materials and methods

### Antibodies and chemicals

The following antibodies were purchased from Cell Signaling Technology (Danvers, MA, USA): rabbit anti-human primary antibodies against CDK2, p21, cleaved caspase-3, caspase-3, cleaved caspase-7, caspase-7, and cleaved caspase-9; mouse anti-human primary antibodies against caspase-9, cyclin A and GAPDH; and HRP-linked secondary antibody (goat anti-rabbit or horse anti-mouse).

DMEM (Dulbecco’s modified Eagle’s medium), penicillin-streptomycin solution, phosphate buffered saline, trypsin and non-fat milk powder were purchased from HyClone (Logan, UT, USA). Fetal bovine serum (FBS) was purchased from Thermo Fisher Scientific Gibco, (Carlsbad, CA, USA). Cell Counting Kit-8 (CCK-8) was purchased from Dojindo Molecular Technology, Inc. (Japan). A propidium iodide (PI) staining kit, RIPA lysate, phenylmethanesulfonyl fluoride (PMSF) and a BCA assay kit were purchased from Beyotime Institute of Biotechnology (Shanghai, China). An Annexin V-FITC/PI double staining kit was purchased from Nanjing Jiancheng Bioengineering Institute (Jiangsu, Nanjing, China). TBST (TBS with 0.05% Tween-20) and ECL reagent were purchased from Beijing Solarbio Science and Technology Co. LTD. (Beijing, China). Bovine serum albumin (BSA), 2,2-diphenyl-1-picrylhydrazyl (DPPH), 2,2ʹ-azino-bis-(3-ethylbenzothiazoline-6-sulphonic acid) (ABTS) and dimethyl sulphoxide (DMSO) were purchased from Sigma-Aldrich Corp (St. Louis, Mo., USA).

Fractions from ‘Noble’ pomace extracts were prepared based on a method described in a previous study [12]. All fractions were dissolved in DMSO. The concentration of fraction 1 (F1) was 30.40 mg of dry weight (DW)/mL, F2 was 16.38 mg DW/mL and F3 was 36.29 mg DW/mL. The composition of each fraction was analyzed by performing LC-TOF-MS/MS at the original concentration indicated above.

F1 contained 1.60 mg/mL kaempferol, 2.09 mg/mL quercetin, 2.47 mg/mL epicatechin, 3.21 mg/mL gallic acid and 1.35 mg/mL ellagic acid (EA). F2 contained 0.65 mg/mL procyanidins and ellagitannins (ET), which resulted in 7.71 mg/mL EA after acid hydrolysis. F3 contained 1.56 mg/mL delphinidin, 0.97 mg/mL cyanidin, 0.95 mg/mL petunidin, 1.07 mg/mL peonidin, 1.20 mg/mL malvidin and 7.19 mg/mL EA. These contents, which were determined by multiplying the fraction concentration (mg DW/mL) by the LC-TOF-MS/MS results, were consistent with the fraction contents in our previous report [13].

### Breast cancer cell lines and culture

The breast cancer cell MDA-MB-231 (the Type Culture Collection of the Chinese Academy of Sciences, China) was cultured in DMEM medium supplemented with 10% FBS and 1% penicillin-streptomycin solution. Cancer cells were incubated in an incubator with 5% CO_2_ at 37°C. The medium was changed every other day.

### Cell viability/inhibition assay

A cell viability assay was performed using the CCK-8 kit following the manufacturer’s protocol. Briefly, 200 µL of medium without cells was added into 5 wells of a 96-well, flat-bottom plate (Corning Life Science, Bedford, MA, USA) as the blank group. For other groups, 200 µL of a single cell suspension at a density of 5.5 × 10^4^ cells/mL was added to the remaining wells of the 96-well plate, which was then incubated in 5% CO_2_ at 37°C. After 24 h, serial concentrations of each fraction were added into each well for each treatment group, while DMSO alone was added to the control group. The final DMSO concentration was 0.25%. After another 24-h incubation period, the medium was removed from all cells, the cells were washed with PBS, and medium containing 10% CCK solution was added into each well. After incubation at 37°C for 3 h, the absorbance was read at 450 nm using a Thermo Multiskan Ascent Reader. The concentrations for all fractions were determined in quintuplicate, and each assay was repeated three times.

The inhibitory rate was calculated using the following formula based on cell viability:

$$Inhibition\;rate\;\left(\%\right)=100\%\;-cell\;viability\;\left(\%\right)=\left[\left(Ac-At\right)/\left(Ac-Ab\right)\right]\times 100\%$$

where Ac is the absorbance of the control group, At is the absorbance of the treatment group, and Ab is the absorbance of the blank group.

### Measurement of anti-oxidative activity

DPPH, ABTS radical scavenging and ferric-reducing antioxidant power (FRAP) assays were carried out according to procedures described in previous studies [14,15]. The absorbance was read at 515, 734 and 593 nm, respectively. For the DPPH and ABTS assays, the inhibition rate of the test sample was calculated using the following formula:

$$Inhibition\;\left(\%\right)=\left(A_{0}-A\right)/A_{0}\times \;100\%$$

where A_0_ was the absorbance of blank (which contained only solvent), and A was the absorbance of the samples.

A calibration curve was established using the inhibition rate and known Trolox solution. All results from these 3 assays were expressed as Trolox equivalent anti-oxidant capacity (µM TE/mL).

### Cell cycle arrest assay

This assay was performed using a PI staining kit following the manufacturer’s instructions. Briefly, cells from the 24-h treatment group or the control group were harvested. After trypsin digestion and centrifugation at 1000 g for 8 min, the supernatants were removed, and the remaining cells were washed with pre-cooled PBS solution. The supernatants were discarded after centrifugation at 1000 g for 10 min. Fixation was performed by adding 70% pre-cooled ethanol to single cells and incubating at −20°C overnight. The ethanol was removed via centrifugation (at 1000 g for 10 min), and the fixed cells were washed with pre-cooled PBS solution followed by centrifugation at 1000 g for 10 min. The supernatants were discarded, and the reaction mixture from the kit was added, followed by incubation at 37°C for 30 min. The assay results were measured using a flow cytometer and analyzed using Mod Fit LT v 3.2.

### Cell apoptosis assay

This assay was performed using the Annexin V-FITC/PI double staining kit per the manufacturer’s instructions. A single-cell suspension from the treatment group or the control group was prepared via trypsin digestion, centrifugation and pre-cooled PBS washing. The supernatant was discarded, and binding solution was added, followed by addition of Annexin V-FITC solution and PI solution. The mixture was incubated at room temperature for 10 min in the dark. The assay results were detected by flow cytometry (FCM) and analyzed using FCS Express v 3.0.

### Western blot

A total of 2 mL of cell suspension with a density of 5.5 × 10^4^ cells/mL was incubated in a 6-well, flat-bottom plate in 5% CO_2_ at 37°C for 48 h. Cells were then treated with 3 different fractions at different concentrations and incubated for another 24 h. Cells were lysed in RIPA lysate buffer containing 1 mM PMSF, which had been used in many other reports [16–18]. The cell lysates were centrifuged, the supernatants were collected and protein concentration was quantified using a BCA assay kit. The concentrations of total protein were normalized based on the results of the BCA assay. The same volume of cell lysate was resolved on a 12% SDS-PAGE gel, followed by trans-blotting onto nitrocellulose membranes. After trans-blotting, membranes were blocked in TBST containing 5% non-fat milk. The blocked membranes were then incubated with properly diluted primary antibodies in TBST containing either 5% non-fat milk or 5% BSA (following the instructions provided) at 4°C overnight. The membranes were then washed three times with TBST and incubated in appropriately diluted HRP-linked secondary antibodies in TBST containing 5% non-fat milk at room temperature for 1 h. Membranes were washed with TBST another three times and visualized using ECL reagent.

### Statistical analysis

Results were expressed as the mean ± standard deviation. All data were statistically analyzed by one-way ANOVA using SPSS v20.0. Significant differences were determined when *p *< 0.05 (Duncan’s test).

## Results and discussion

### Determination of cell inhibitory concentrations among fractions

**Figure 1.**
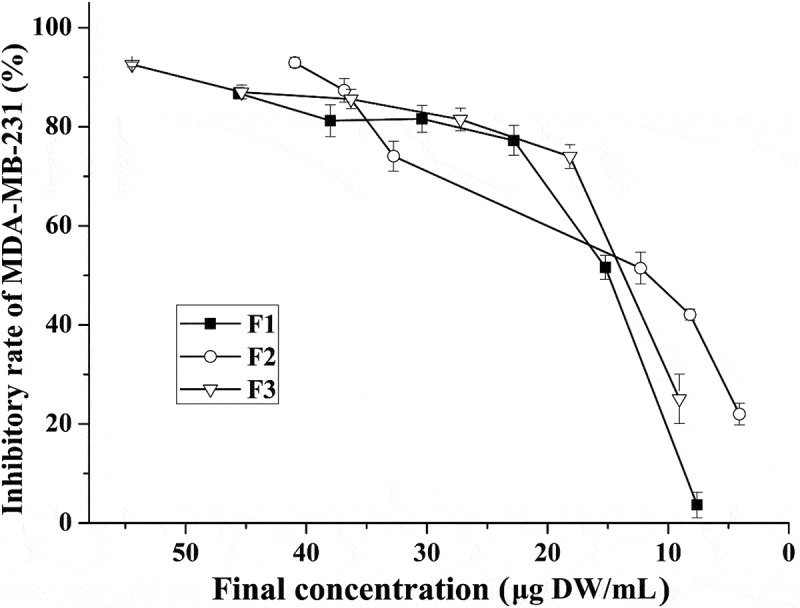
Inhibition rates for MDA-MB-231 cells after treatment with isolated fractions of ‘Noble’ pomace. F1: mixture of flavonoids, phenolic acids and EA; F2: mixture of tannins; F3: mixture of anthocyanidins and EA.

A pilot study had been done to confirm the solvent (0.25% DMSO) did not affect the cell viability. After treating cancer cells with each fraction extracted from ‘Noble’ pomace, the highest growth inhibition rates were 86.7%, 92.9% and 92.6% for F1, F2 and F3, respectively (Figure 1). Two different cell inhibitory concentrations, IC_80_ (80% maximal inhibitory concentration) and IC_50_ (50% maximal inhibitory concentration), of each fraction were calculated based on the inhibition curve. The IC_80_ and IC_50_ values of F2 were 20.85 ± 2.37 and 9.75 ± 0.52 µg DW/mL, respectively. These values were significantly lower than those of F1 in which the IC_80_ was 29.64 ± 1.50 µg DW/mL and the IC_50_ was 17.82 ± 2.42 µg DW/mL, but they were similar to those for F3, which was 25.00 ± 1.73 and 12.88 ± 2.69 µg DW/mL for IC_80_ and IC_50_, respectively (Table 1).

**Table 1.** 80% and 50% maximum inhibitory concentrations of different fractions.

Group	Concentration (µg DW/mL)	
	IC_50_ (low concentration)	IC_80_ (high concentration)
F1	17.82 ± 2.42^a^	29.64 ± 1.50^a^
F2	9.75 ± 0.52^b^	20.85 ± 2.37^b^
F3	12.88 ± 2.69^ab^	25.00 ± 1.73^ab^

F1: mixture of flavonoids, phenolic acids and EA; F2: mixture of tannins; F3: mixture of anthocyanidins and EA. The same letter in the same column indicates no significant differences (*p *≥ 0.05).

Lower IC_50_ and IC_80_ values indicated a stronger capacity for cell growth inhibition. Based on the above results, similar IC_50_ and IC_80_ values were obtained for F2 and F3. Thus, identifying the most effective fraction only by comparing the values of these two inhibitory concentrations was not sufficient, the anti-oxidative activity as well as the ability to induce cell cycle arrest and apoptosis which were used for the evaluation of the anti-cancer function exerted by phenolic compounds [11], should also be compared after treating the cells with IC_80_ (high concentration) and IC_50_ (low concentration).

### Anti-oxidative activities among fractions

The anti-oxidative activities among the 3 fractions varied in both low and high concentrations (Table 2). In DPPH assay, F3 showed a value of 4.44 ± 0.38 and 2.33 ± 0.18 µM TE/mL for high and low concentrations, and both of these figures were the highest values among all fractions. No difference was found in the anti-oxidative activities between F1 and F2. Similarly in FRAP assay, F3 presented a value of 5.87 ± 0.09 µM TE/mL for high and 2.94 ± 0.05 µM TE/mL for low concentration treatments, which also indicated the strongest anti-oxidative activity among the three fractions. However, at both concentrations, the results from the ABTS assay did not significantly differ among all the fractions. Different values are obtained for different assays because each method has multiple reaction characteristics and mechanisms [19]. Therefore, to obtain a more accurate picture of anti-oxidative activity, it is preferable to evaluate activity in multiple assays.

**Table 2.** Anti-oxidative activities of different fractions at low and high concentrations.

	DPPH (µM TE/mL)		ABTS (µM TE/mL)		FRAP (µM TE/mL)	
	Low conc.	High conc.	Low conc.	High conc.	Low conc.	High conc.
F1	1.34 ± 0.20^b^	2.27 ± 0.34^b^	2.33 ± 0.25^a^	3.95 ± 0.42^a^	2.37 ± 0.21^b^	4.01 ± 0.36^b^
F2	1.24 ± 0.31^b^	2.63 ± 0.65^b^	1.91 ± 0.42^a^	4.06 ± 0.90^a^	2.16 ± 0.06^b^	4.60 ± 0.13^b^
F3	2.23 ± 0.18^a^	4.44 ± 0.38^a^	2.57 ± 0.14^a^	5.13 ± 0.27^a^	2.94 ± 0.05^a^	5.87 ± 0.09^a^

Low conc.: low concentration; High conc.: high concentration. F1: mixture of flavonoids, phenolic acids and EA; F2: mixture of tannins; F3: mixture of anthocyanidins and EA. The same letter in the same column indicates no significant differences (*p *≥ 0.05).

### Ability to induce cell cycle arrest as determined by FCM

**Figure 2.**
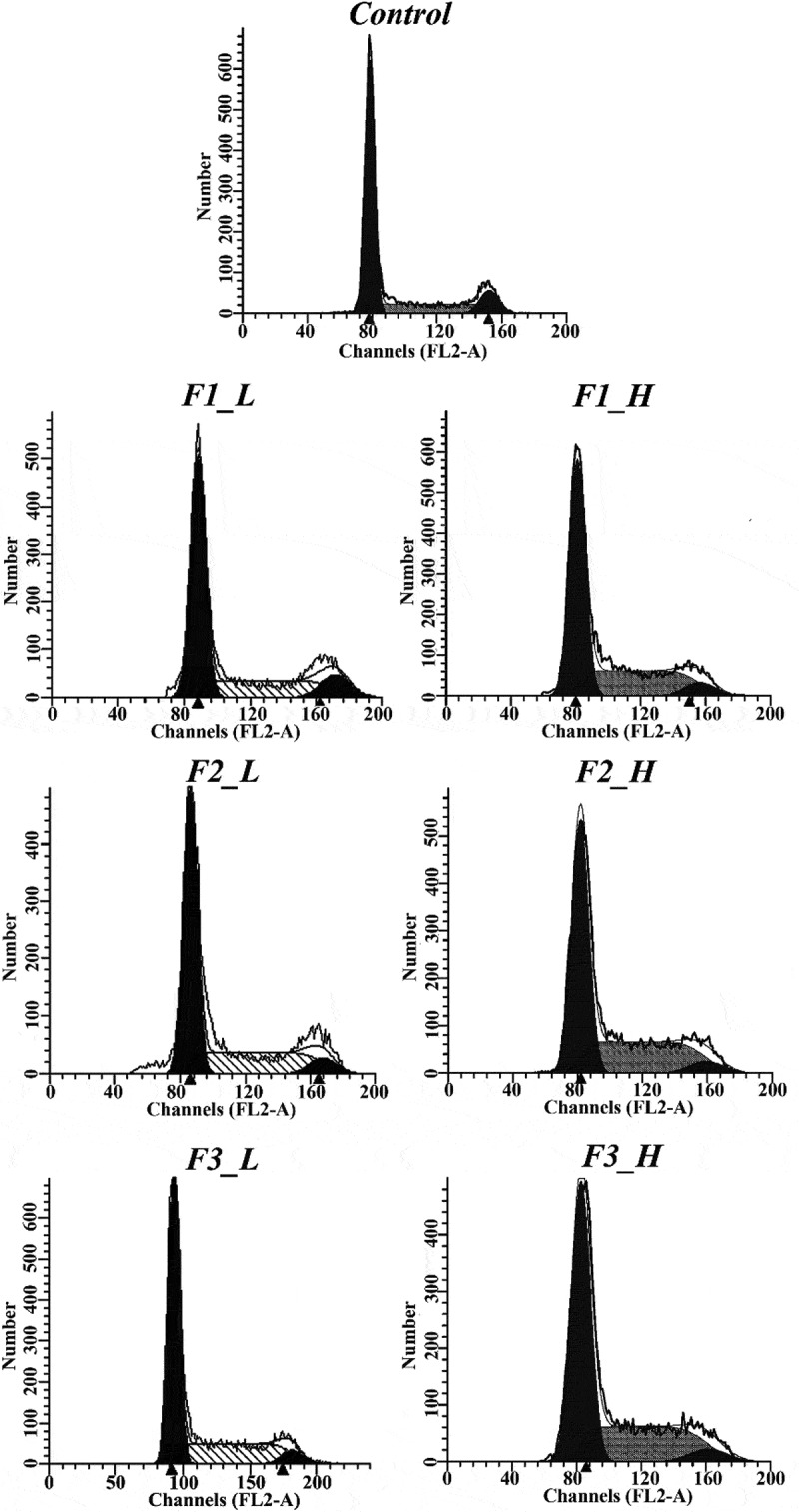
Plots from the cell cycle arrest assay using different fractions at low and high concentrations. Control: solvent treatment; F1 (F2, F3)_L: low concentrations of F1 (F2, F3); F1 (F2, F3)_H: high concentrations of F1 (F2, F3).

After treating cells with fractions of ‘Noble’ pomace extract in either low or high concentration, proportions of cell populations in the G0/G1, S and G2/M phases changed drastically in each group at varied extents (Figure 2).

Treatment with low concentrations of F1, F2 and F3 caused 30.28%, 34.13% and 33.16% of cells to be arrested in S phase, while in the control there were 18.22% cells in the S phase. These results indicate that F1 induced the lowest amount of S phase arrest, while F2 and F3, with no difference between them, resulted in higher percentages of cells arrested in S phase. In high concentrations, treatment with F1 resulted in a significantly higher proportion (36.88%) of cells retarded in S phase than the control (18.22%), and even stronger S phase arrests were found in F2 and F3 treatments where 40.28% and 39.66% of cells were trapped in S phase (Table 3).

**Table 3.** Proportion of cells in different cell cycle phases after treatment with different fractions at high and low concentration (mean ± standard deviation).

Phase	Concentration	Group			
		Control	F1	F2	F3
G0/G1	Low	67.17 ± 0.52^a^	59.50 ± 0.21^b^	58.49 ± 0.13^c^	60.25 ± 0.13^b^
	High	67.17 ± 0.52^a^	56.90 ± 0.34^b^	54.21 ± 1.50^c^	55.20 ± 0.06^bc^
S	Low	18.22 ± 0.07^c^	30.28 ± 0.22^b^	34.13 ± 0.38^a^	33.16 ± 0.71^a^
	High	18.22 ± 0.07^c^	36.88 ± 0.21^b^	40.28 ± 0.80^a^	39.66 ± 0.13^ab^
G2/M	Low	14.61 ± 0.48^a^	10.22 ± 0.64^b^	7.38 ± 0.31^c^	6.58 ± 0.22^c^
	High	14.61 ± 0.48^a^	6.32 ± 0.04^b^	5.52 ± 0.63^b^	5.15 ± 0.17^b^

F1: mixture of flavonoids, phenolic acids and EA; F2: mixture of tannins; F3: mixture of anthocyanidins and EA. The same letter in the same row indicates no significant differences (*p *≥ 0.05).

Based on the FCM measurement on cell cycle arrests, fraction F2 had the strongest ability to cause S phase arrest, followed by F3 and F1. Interestingly, the ability to induce cell cycle arrest in S phase coincides with the EA content in each fraction. Although F2 is primarily composed of ETs, it was previously demonstrated that ETs must first be hydrolyzed into EA to be functional and cause S phase arrest in prostate cancer cells [20]. According to the results of our current study, as well as those in the previous report, the ability to induce cell cycle arrest may be attributable to the EA content within each fraction. Notably, there was a similar effect exerted on S phase arrest by the F2 and F3 fractions at both concentrations, also indicating that treatment with the F3 fraction efficiently induces cell cycle arrest.

### Comparison of cell cycle arrest by Western blot analysis

**Figure 3.**
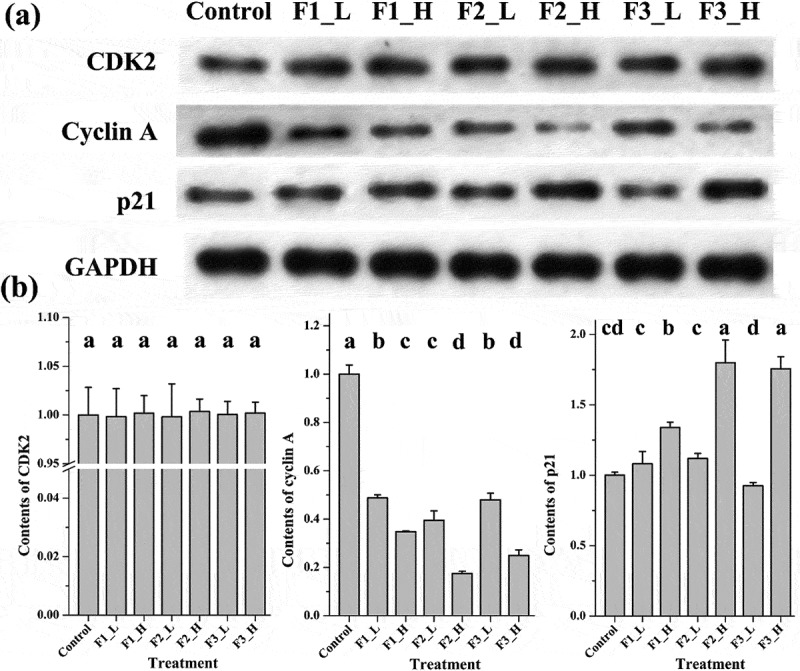
Expression of cell cycle arrest proteins following treatment with different fractions at high or low concentrations. (a) Bands obtained from Western blot analysis; (b) bar charts of the mean integrated density of each band over the corresponding integrated density of GAPDH (control value adjusted to 1). ‘a, b, c’: the same letter above the bars indicates no significant differences (*p *≥ 0.05). Control: solvent treatment; F1 (F2, F3)_L: low concentrations of F1 (F2, F3); F1 (F2, F3)_H: high concentrations of F1 (F2, F3).

Cyclin A, CDK2 and p21 were tested to determine which fraction presented the strongest ability to induce cell cycle arrest at the protein level. As shown in Figure 3, after treating the cells with muscadine fractions at low concentrations, cyclin A expression levels in all treatment groups were at least 2 times lower than those in the control group, as measured by the integrated densities of Western blot bands. Among them, the treatment with F2 induced significantly lower expression of cyclin A than treatment with F3 and F1, which exerted similar effects on cyclin A down-regulation. At high concentrations, treatment with all fractions, and particularly F2 and F3, induced much stronger down-regulation of cyclin A expression. Cyclin A down-regulation was affected in the following order: F2 exerted the strongest effects, followed by F3 (similar to F2 at high concentration) and then F1. Additionally, at low concentrations, no statistical differences were observed on expression of the p21 protein among three treatments. But at high concentrations, different treatments induced different expressing levels of p21 protein. A higher level of p21 protein was obtained in treatments F2 and F3 than in treatment F1. This result strongly supports the FCM cell cycle analysis. Expression of the cytokine CDK2, on the other hand, remains unchanged in all treatment groups regardless of the fraction concentrations.

Formation of the CDK2/cyclin A complex is crucial for cells to pass through S phase and enter G2/M phase [21]. Therefore, the down-regulation of either cytokine causes cells to accumulate in S phase, resulting in S phase arrest. The cytokine p21 is an inhibitor of CDK2 and binds to the CDK2/cyclin E complex to induce G1 arrest [22–25], but it has also been reported to induce S phase arrest [26]. Since no significant changes in CDK2 expression was measured in the current study, the ability of F2 and F3 to induce the down-regulation of cyclin A and up-regulation of p21 expression especially at high concentration made it the most effective fraction for inducing S phase arrest.

### Ability to induce apoptosis as determined by FCM

**Figure 4.**
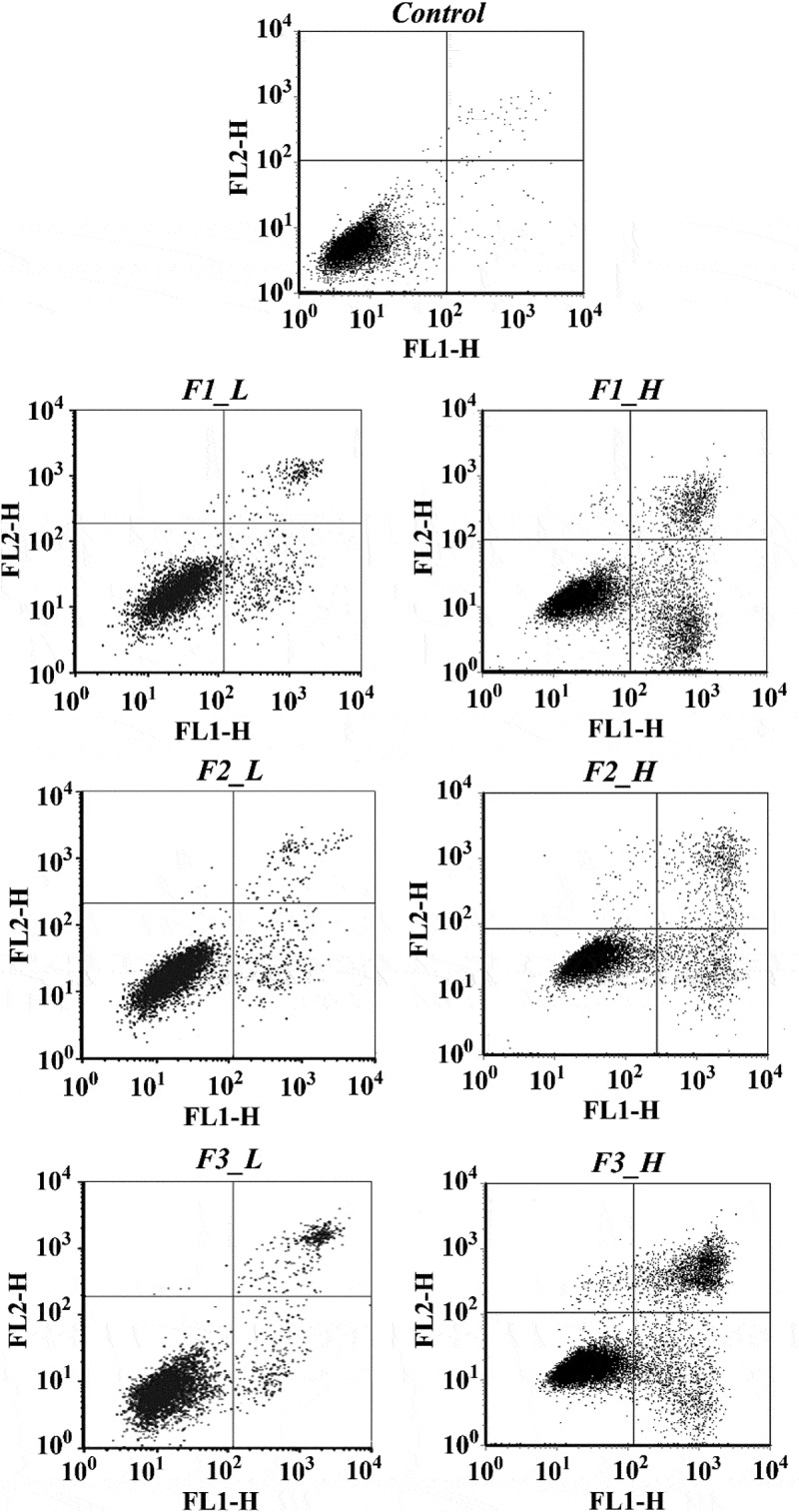
Plots from the cell apoptosis assay for different fractions at low and high concentrations. Control: solvent treatment; F1 (F2, F3)_L: low concentrations of F1 (F2, F3); F1 (F2, F3)_H: high concentrations of F1 (F2, F3).

Following treatments of cells with fractions of ‘Noble’ pomace extracts in either low or high concentrations, the proportions of normal and apoptotic cells varied (Figure 4). Only 0.40% of the total cells were apoptotic in the control group, while treatments with F1, F2 and F3 at low concentrations increased the apoptotic cells by 9.63%, 6.07% and 11.63%, respectively. Higher percentages of apoptotic cells were observed in all 3 fractions in the treatments with higher concentrations (Table 4). In general, treatment with fraction F3 induced the significantly highest numbers of apoptotic cells in both concentrations, followed by F1 and then F2.

**Table 4.** Proportions of normal and apoptotic cells after treatment with different fractions at high and low concentrations (mean ± standard deviation).

Cell	Concentration	Group			
		Control	F1	F2	F3
Normal	Low	99.24 ± 0.17^a^	89.85 ± 0.45^c^	93.45 ± 0.32^b^	87.91 ± 0.13^d^
	High	99.24 ± 0.17^a^	79.17 ± 0.11^c^	87.15 ± 0.11^b^	77.14 ± 0.16^d^
Apoptotic	Low	0.40 ± 0.08^d^	10.03 ± 0.37^b^	6.47 ± 0.63^c^	12.03 ± 0.63^a^
	High	0.40 ± 0.08^d^	19.63 ± 0.48^b^	11.99 ± 0.50^c^	21.52 ± 0.58^a^

F1: mixture of flavonoids, phenolic acids and EA; F2: mixture of tannins; F3: mixture of anthocyanidins and EA. The same letter in the same row indicates no significant differences (*p *≥ 0.05).

F3 is primarily composed of monomers with high bioavailability, such as anthocyanins, while fraction F2 contains polymers such as ETs and procyanidins. ETs belong to the family of hydrolysable tannins, which are hydrolyzed into EA, but the procyanidins, which belong to the condensed tannin family, are stable and have relatively low bioavailability [27]. Therefore, the ability to induce apoptosis is more likely to be related to the bioavailability of phenolic compounds.

### Comparison of apoptosis induction by Western blot analysis

**Figure 5.**
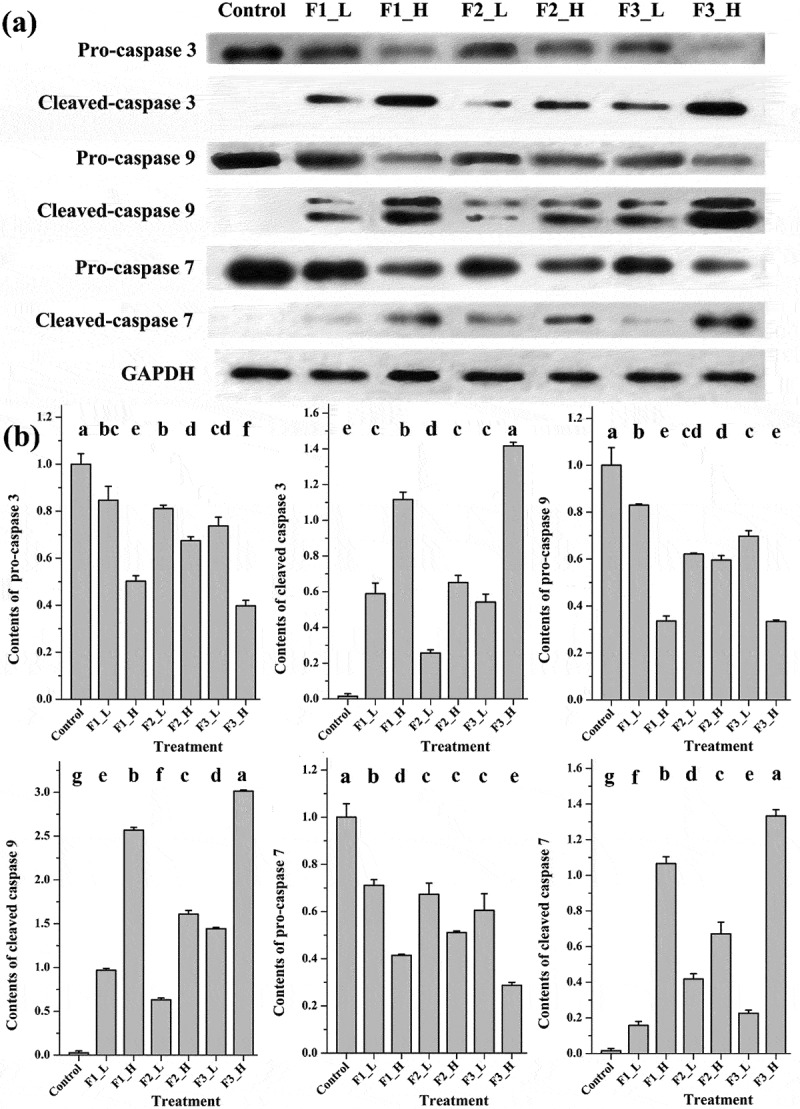
Contents of apoptosis proteins following treatment with different fractions at high or low concentrations. (a) Bands obtained from Western blotting; (b) bar charts of the mean integrated density of each band over the corresponding integrated density of GAPDH (control value adjusted to 1). ‘a, b, c’: the same letter above the bars indicates no significant differences (*p *≥ 0.05). Control: solvent treatment; F1 (F2, F3)_L: low concentrations of F1 (F2, F3); F1 (F2, F3)_H: high concentrations of F1 (F2, F3).

Caspases, an important group of enzymes belonging to the cysteine protease family, are crucial for apoptosis. The cleavage of pro-caspase into cleaved caspase indicates the activation and initiation of degradation of chromosomal DNA as well as nuclear and cytoskeletal proteins [28]. Three caspase family members, caspase-3, -9 and -7, either in their pro- or cleaved forms, were detected by Western blot to determine which fraction had the strongest ability to induce apoptosis at the protein level. As shown in Figure 5, the expression of pro-caspase-3 was reduced while the corresponding cleaved proteins were raised significantly after treatment with F3 at both low and high concentrations. This result indicated that F3 could activate the caspase-3, and the higher of the treating concentration, the more activated form of caspase-3 was found. Similar results were also obtained from caspase-9 and caspase-7. The treatment with F2 and F1 also activated 3 types of caspases, but much weaker effect was observed.

Caspase-3 is the most important executioner caspase, directly activating associated endonucleases and proteases and leading to the degradation of chromosomal DNA, as well as nuclear and cytoskeletal proteins [28]. Caspase-9 lies upstream of caspase-3 and is considered the key initiator caspase of the intrinsic apoptotic pathway [29]. Moreover, caspase-7 is a type of executioner caspase with high similarity to caspase-3 [30]. It has been reported that, both anthocyanidins and ellagic acid induced apoptosis in multiple types of cancer cell lines via activating the caspases [31–34]. Therefore, the increased cleavage of all 3 caspases following treatment with F3, which contained a mixture of anthocyanidins and ellagic acid, would result in its strongest ability for apoptosis induction.

## Conclusion

Based on the results of the present study, fraction F3, which contained a mixture of anthocyanidins and EA, exhibited the strongest anti-oxidative activity. In comparison with F1 and F2 fractions, F3 also exhibited the strongest ability to induce apoptosis and cell cycle arrest. This was likely attributable to the ability of F3 more effectively activating caspase-3, -7 and -9, and more efficiently inducing S phase arrest by down-regulating the expression of cyclin A and up-regulating the expression of p21. The results indicated that F3 is the most effective fraction in breast cancer cell MDA-MB-231 inhibition among the three fractions prepared from muscadine grape ‘Noble’ pomace.
